# Chasing the fitness optimum: temporal variation in the genetic and environmental expression of life-history traits for a perennial plant

**DOI:** 10.1093/aob/mcad100

**Published:** 2023-07-26

**Authors:** Mason W Kulbaba, Zebadiah Yoko, Jill A Hamilton

**Affiliations:** Our Lady of the Lake University, Department of Mathematics and Science, San Antonio, TX 78207, USA; St Mary’s University, Biology Area, 14500 Bannister Road SE, Calgary, Alberta, Canada, T2X 1Z4; North Dakota State University, Department of Biological Sciences, Fargo, ND 58102, USA; North Dakota State University, Department of Biological Sciences, Fargo, ND 58102, USA; Pennsylvania State University, Department of Ecosystem Science and Management, University Park, PA 16801, USA

**Keywords:** Life history, quantitative genetics, fitness, phenology, fitness landscape, climate change, *Geum triflorum*, alvar habitat, prairie habitat, common garden

## Abstract

**Background and Aims:**

The ability of plants to track shifting fitness optima is crucial within the context of global change, where increasing environmental extremes may have dramatic consequences for life history, fitness, and ultimately population persistence. However, tracking changing conditions relies on the relationship between genetic and environmental variance, where selection may favour plasticity, the evolution of genetic differences, or both depending on the spatial and temporal scale of environmental heterogeneity.

**Methods:**

Over three years, we compared the genetic and environmental components of phenological and life-history variation in a common environment for the spring perennial *Geum triflorum*. Populations were sourced from alvar habitats that exhibit extreme but predictable annual flood–desiccation cycles and prairie habitats that exhibit similar but less predictable variation in water availability.

**Key Results:**

Heritability was generally higher for early life-history (emergence probability) relative to later life-history traits (total seed mass), indicating that traits associated with establishment are under stronger genetic control relative to later life-history fitness expressions, where plasticity may play a larger role. This pattern was particularly notable in seeds sourced from environmentally extreme but predictable alvar habitats relative to less predictable prairie environments. Fitness landscapes based on seed source origin, largely characterized by varying water availability and flower production, described selection as the degree of maladaptation of seed source environment relative to the prairie common garden environment. Plants from alvar populations were consistently closer to the fitness optimum across all years. Annually, the breadth of the fitness optimum expanded primarily along a moisture gradient, with inclusion of more populations onto the expanding optimum.

**Conclusions:**

These results highlight the importance of temporally and spatially varying selection in life-history evolution, indicating plasticity may become a primary mechanism needed to track fitness for later life-history events within perennial systems.

## INTRODUCTION

The timing of environmental cues essential to life-history transitions may shift as a consequence of global climate change, increasing the potential discrepancy between realized and optimal fitness (Reed *et al.*, 2010; Edelaar *et al.*, 2017). Optimizing fitness requires a combination of strategies, including the evolution of locally adapted genotypes that are the product of natural selection (Clausen *et al.*, 1941; Kawecki and Ebert, 2004; Leimu and Fischer, 2008; Hereford, 2009) and phenotypic plasticity, and the ability of individual genotypes to directly alter phenotypes in response to the environment (Bradshaw, 1965; Scheiner, 1993; Josephs, 2018). As environments change, ensuring individuals have the capacity to maximize fitness through different strategies is crucial to population persistence. However, the relative degree to which adaptive evolution and plasticity evolve can vary across traits, particularly if different life-history stages disproportionately mediate the fitness effects of environmental heterogeneity (Roff and Mousseau, 1987). Within the context of climate change, increased environmental heterogeneity and decreased temporal and spatial predictability of environmental cues may directly impair the ability of individuals to track fitness optima via adaptive evolution and plasticity (Aitken *et al.*, 2008; Baythavong, 2011; Franks *et al.*, 2013; Anderson and Gezon, 2015). Consequently, it is essential to quantify the impact that spatially and temporally varying environments may have on adaptation and plasticity for life-history traits and long-term fitness.

Realized phenotypes are the combined products of genetic and environmental effects, with fitness as an emergent property determined by the similarity between individual genotypes and environmentally determined optima. With this two-fold contribution, limited variation in heritable effects or plastic responses to environmental conditions can constrain the match between optimal and realized phenotypes, ultimately impacting fitness. The relative amounts of phenotypic variation attributed to heritable and plastic components will differ both among traits (Mousseau and Roff, 1987; Price and Schluter, 1991) and environments (Hoffmann and Merila, 1999). For example, phenological schedules require at least some level of genetic determination to initiate life-history events, but plasticity is often required to achieve maximum fitness given the variability inherent to natural environments. Theory suggests that the proportion of phenotypic variation attributed to plasticity should be greater in environments with repeated and predictable cues compared with more unpredictable environments (Lande, 2009; Botero *et al.*, 2015; Tufto, 2015; Leung *et al.*, 2020). Starrfelt and Kokko (2012) identified strategies that organisms employ to contend with environmental uncertainty. When environmental fluctuations are unpredictable, individuals may employ a generalist strategy to minimize fitness variation in response to fluctuating environments (Starrfelt and Kokko, 2012). This may enable persistence across a range of environments, but may lead to reduced overall fitness as a conservative bet-hedging strategy. When environmental cues are predictable, organisms may favour adaptive plasticity as a means to adjust phenotypes in response to the environment to maximize fitness (Scheiner, 2013). However, plasticity may be constrained when there is limited capacity (Auld *et al.*, 2010) or the maintenance and expression of plasticity is costly (DeWitt *et al.*, 1998). Adaptive plasticity may also evolve in traits closely linked to fitness to both maintain fitness under unfavourable conditions and maximize fitness when conditions are more favourable (Sultan, 2001). With expected increases in global environmental variability and decreased predictability of cues under climate change (Botero *et al.*, 2015), the likelihood of potential mismatches between phenotypes and environments is increasing. This trend highlights the need to refine our understanding of variation in the genetic and environmental components contributing to life-history evolution across spatially and temporally varying environments.

A major challenge to predicting responses to environmental change is the complexity of interacting effects. This is particularly evident where exposure to varying degrees of micro-environmental variation influence a population’s adaptive capacity (Denney *et al.*, 2020). Quantitative genetic theory predicts that trait heritability varies across environments and time (Price and Schluter, 1991; Lynch and Walsh, 1998) and environmental heterogeneity can promote the evolution of plasticity across a species’ range (Kingsolver *et al.*, 2002; Baythavong, 2011; Scheiner, 2013; Edelaar *et al.*, 2017). Selection for plasticity should occur in environments with predictable cues, such as those marking seasonal transitions. Such consistent selection may result in reduced genetic variation for plasticity (Oostra *et al.*, 2018). This contrasts with expectations for populations sourced from unpredictable environments, where genetic variation in plastic responses is expected to be maintained, enhancing trait plasticity (Ghalambor *et al.*, 2007; Chevin *et al.*, 2010). Given these contrasting predictions, understanding the degree to which environmental heterogeneity and plasticity interact to influence phenotypic variation is essential to predicting population fitness over space and time. However, quantifying adaptive evolution in part requires detailed estimates of the degree of constancy for quantitative genetic parameters across time and environments (Young *et al.*, 1994; Bemmels and Anderson, 2019). Indeed, genetic variation for plasticity may generate changes in adaptive genetic variance across environments; therefore, genetic and environmental responses may not be mutually exclusive. Quantifying the relative influence of genetic and environmental variation on life-history traits in natural settings is difficult as the consistency of environmental cues cannot be experimentally controlled. Thus, the use of ecotypic variants in common garden experiments can facilitate estimation of quantitative parameters across space and time.

In the present study, we test the prediction that individuals sourced from predictably heterogeneous environments will exhibit greater heritability for life-history traits relative to those from less predictable, but still heterogeneous environments. In addition to environment-specific variation in heritability, we expect that the heritability of traits varies with ontogeny. Specifically, we predict that early life-history traits, such as emergence, will be under greater genetic control relative to later life-history stages, representing a continuum of relatively heritable to more plastic traits. Few studies have quantified the relative genetic and environmental contributions to life history in perennial plant species due to challenges associated with estimating lifetime fitness (but see Campbell, 1996, 1997; Simons and Johnston, 2000). However, these data remain key to quantifying the capacity of long-lived species to persist across temporal and spatially varying fitness landscapes. Finally, we evaluate individuals’ ability to traverse the fitness landscape following an experimental increase in distance between a home and novel common garden environment. Quantifying the impact that environmental predictability may have on the evolution of life history and the ability of species to compensate for mismatches across life-history stages will be essential to predicting the capacity for species to track changing conditions and modify life-history strategies under global change.

We quantified temporal and spatial genetic variances for traits associated with life history and fitness in the perennial forb *Geum triflorum* sourced from distinct habitats with contrasting predictability of environmental cues. *Geum triflorum* is an early-season, spring perennial common to Midwestern prairie habitats generally characterized by cold, dry winters and hot, humid summers that experience shifts in water availability both annually and seasonally that are relatively unpredictable (Hamilton and Eckert, 2007; Yoko *et al.*, 2020; Volk *et al.*, 2022). This contrasts with populations of *G. triflorum* persistent on alvar habitats isolated throughout the Great Lakes region of North America. Alvar habitats experience extreme but predictable annual seasonal variation in water availability from complete flooding in early spring to complete desiccation by early summer (Catling and Brownell, 1995; Stark *et al.*, 2004; Yoko *et al.*, 2020; Volk *et al.*, 2022). Specifically, we ask (1) across the sequential continuum of life-history events, is there variance in the heritability of life-history traits and expressions of fitness, and if so, are there consequences to lifetime fitness? (2) Do estimates of evolvability for life-history traits and expressions of fitness indicate the potential for genotypes to become locally adapted to the common garden environment? and (3) Does the heritability of phenology and life-history events impede the ability of individuals to maximize proximity to the optimum of the fitness landscape? Understanding how spatially and temporally varying selection interacts with heritable genetic and environmental components across species’ life history will be essential to predicting fitness under global change.

## MATERIALS AND METHODS

### Study site and species

**Fig. 1.**
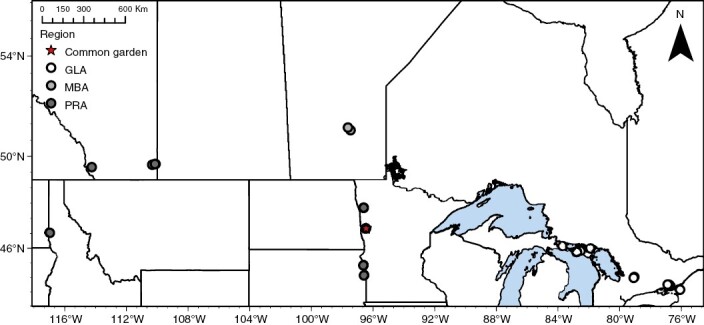
Map indicating location of common garden and Great Lakes alvars (GLA), Manitoba alvars (MBA) and prairie (PRA) source populations of *G. triflorum*.

This study focuses on *Geum triflorum*, commonly known as prairie smoke, a widespread perennial forb from the Rosaceae family. Prairie smoke is largely distributed across native prairie habitat throughout the Great Plains of North America but is also common on geographically disjunct alvar habitats dispersed around the Great Lakes and into Manitoba, Canada. Open-pollinated maternal seed families were collected in the spring of 2015 from 19 populations of *G. triflorum*, including 11 populations from the Great Lake alvar region, two from the Manitoba alvar region, and six from the prairie region of the Midwest (Yoko *et al*, 2020). Within each population ~40 seed families were collected along a 100-m transect (as in Hamilton and Eckert, 2007). To supplement these collections, three additional bulk seed collections for prairie populations were obtained from commercial seed providers (SD-PMG, MN-PMG) and the USDA-Pullman Plant Materials Center (WA-BLK) (Fig. 1). Alvar (Great Lakes and Manitoba) and prairie habitats differed primarily in terms of within-year water availability (Catling and Brownell, 1995; Stark *et al.*, 2004), but also demographically within the alvar sites. For example, the Great Lakes alvar populations exist as large and dense populations compared with the Manitoba alvar populations (Hamilton and Eckert, 2007).

On 7 November 2015 a common garden experiment was established using open-pollinated seeds at North Dakota State University (described in Yoko *et al*., 2020). Using a randomized complete block design, ten maternal seed families were planted for each of the 19 populations across 12 blocks, including 12 individual half-sibs per maternal family (*n* = 2280). For the three bulk populations, two replicates were planted across each of the 12 blocks, for a total of 24 seeds per bulk population collection (*n* = 72). In total, 2352 individual seeds treated with 0.02 % PPM fungicide were planted in ‘Cone-tainers’ (158 mL, Stuewe & Sons) filled with Sungro horticulture mix (1N-45P-12K) soil in a greenhouse at North Dakota State University (Table 1). The greenhouse was maintained at 15-h days with supplementary daylight from halide lighting at a measured flux density of 0.3383 mmol m^-^^−^^2^ s^−1^ for the duration of the experiment and temperatures fluctuating between 18.3 and 23.9°C. Seedlings were watered twice weekly and provided with a slow-release fertilizer mix (Osmocote 14N-14P-14K) throughout the course of the experiment. In May 2016, surviving seedlings were transferred to a permanent outdoor research facility at the Minnesota State University at Moorhead Regional Science Center (46.86913, −96.4522). Individuals were planted directly into the ground through cut-outs in a weed barrier to limit competition. The randomized block design established in the greenhouse was maintained at the permanent field site.

**Table 1.** Source populations of *G. triflorum* collected in 2015 spanning three distinct eco-regions (GLA, Great Lakes alvars; MBA, Manitoba alvars; PRA, prairie), along with latitude, longitude and elevation (m) of population collection sites. Distance from common garden experiment (km) notes the greater circle distance calculated between the population origin and the common garden experiment established at Minnesota State University, Moorhead Regional Science Center, Moorhead, Minnesota, USA.

Population ID	Region	Latitude	Longitude	Elevation (m)	Distance from common garden experiment (km)
CAR-NBA	GLA	44.69	−79.05	268	1368
CAR-PSR	GLA	44.65	−79.09	250	1366
MAN-FOX	GLA	45.90	−82.58	186	1068
MAN-KIP	GLA	45.87	−82.54	183	1072
MAN-LCI	GLA	45.99	−81.89	182	1118
MAN-MIS	GLA	45.81	−82.76	193	1056
MI-DRI	GLA	46.09	−83.69	188	980
NAP-ASS	GLA	44.27	−76.71	126	1559
NAP-CE	GLA	44.33	−76.79	166	1551
NAP-SCH	GLA	44.34	−76.89	154	1543
WNY-CB	GLA	44.10	−76.08	93	1613
MB-CRN	MBA	51.07	−97.46	231	473
MB-MR	MBA	51.18	−97.63	231	487
AB-HSC	PRA	49.64	−110.33	721	1071
AB-LL	PRA	49.54	114.25	929	1348
AB-RL	PRA	49.67	−110.11	721	1056
AB-RO	PRA	49.67	110.15	721	1059
MN-PMG	PRA	47.77	−96.61	267	101
ND-BSP	PRA	46.86	−96.47	274	2
SD-MUD	PRA	44.76	−96.59	531	234
SD-PMG	PRA	45.22	−96.63	351	184
WA-BLK	PRA	46.69	−116.97	786	1558
Common garden experiment	PRA	46.87	−96.45	259	–

### Data collection

Phenological and life-history fitness components were evaluated within the common garden from 2015 to the end of the growing season in 2018. Single-season phenological observations included the number of days from planting to emergence and establishment of true leaves, recorded in 2015. In addition, multi-year observations were taken for number of days between planting and bolting (defined as the initial elongation of the flowering stem to ~7 cm, recorded in 2017 and 2018), days between planting and flowering (recorded annually between 2015 and 2018) and days between planting and the initiation of infructescence development (defined as the date developing woolly styles extend beyond the corolla to form a diaspore for dispersal, recorded in 2017 and 2018).

To capture annual estimates of reproductive output in the common garden, cloth mesh bags (Uline S-13940) were tied and labelled around each individual infructescence as the woolly styles began to extend beyond the corolla. Cloth mesh bags provide the opportunity for the diaspore to fully mature, while limiting potential loss of reproductive output via wind dispersal. Cloth mesh bags were harvested in each August of the monitoring year. Number of reproductive stems, identified as stems with infructescences, was used to quantify the number of fruits produced per individual. Seed mass was taken to reflect potential reproductive output per reproductive stem. Total annual fitness was estimated as the cumulative seed mass produced per individual genotype based on all reproductive stems produced. Seed mass is considered a proxy for the number of seeds produced per individual. In 2016, we performed a regression between the number of seeds produced and seed mass for one reproductive stem per individual planted within the common garden (*R*^2^ = 0.528). Flowering-only stems were also quantified in the field as individuals that had flowered, but senesced prior to producing seed, and thus considered non-reproductive. To quantify the total number of flowers produced per individual within each season we combined the total number of reproductive stems with flowering-only stems. Those individuals that did not produce a flowering-only stem or reproductive (flower + fruit) stems were noted each year as having survived but were classed as completely non-reproductive.

### Statistical analyses

To determine the heritability of life-history and phenological traits, we used generalized linear mixed models (Stroup, 2013) as implemented in the package lme4 (Bates *et al*., 2014) using a maternal half-sibling sampling design (Lynch and Walsh, 1998). These analyses employed the most appropriate sampling distributions and link functions for each trait (Table 2), as determined through comparison of Akaike information criterion (AIC) values across models. All analyses included the effect of block and population as fixed effects, maternal family as a random effect, and the interaction between block and maternal family (a random interaction effect). Following Ahrens *et al*. (2020) we calculated family-level heritability as:

**Table 2.** Narrow-sense heritability (standard errors) for life-history fitness expression (A) and phenological traits (B) of common garden *G. triflorum* plants from three regions.

(A) Life history fitness expression												
	Germination (yes/no)	Number of flowers			Number of fruits			Seed mass				
Region	2015	2016	2017	2018	2016	2017	2018	2016	2017	2016 and 2017	2018	Total
Great Lakes alvar	0.263 (0.030)	0.034^1^ (0.03)	0.016 ^1^ (0.015)	0.024^2^ (0.080)	0.023^2^ (0.032)	0^2^ (na)	0.007^2^ (0.001)	0.001^3^ (0.230)	0^2^ (na)	0^2^ (na)	0^3^ (na)	0^2^ (na)
Manitoba alvar	0.149 (0.080)	0.129^2^ (0.05)	0.025^2^ (0.040)	0.044^2^ (0.040)	0^1^ (na)	0^2^ (na)	0^2^ (na)	0^2^ (na)	0^3^ (na)	0.001^2^ (0.06)	0^2^ (na)	0^2^ (na)
Prairie	0.112 (0.062)	0^1^ (na)	0.034^2^ (0.042)	0.122^2^ (0.030)	0^2^ (na)	0.001^2^ (0.030)	0^2^ (na)	0^3^ (na)	0^2^ (na)	0^2^ (na)	0^2^ (na)	0^2^ (na)

(B) Phenological traits												
	Planting to germination	Planting to first true leaf	Germination to first true leaf	Planting to bolting		Planting to first flower			Planting to fruit set			
Region	2015	2015	2015	2017	2018	2016	2017	2018	2017	2018		
Great Lakes alvar	0.213^1^ (0.040)	0.253^1^ (0.041)	0.099^1^ (0.012)	0.124^1^ (0.107)	0.039^1^ (0.038)	0.050^1^ (0.41)	0.084^1^ (0.04)	0.082^2^ (0.06)	0.001^1^ (0.001)	0.018^1^ (0.033)		
Manitoba alvar	0^2^ (na)	0.130^2^ (0.111)	0.023^2^ (0.057)	0.034^1^ (0.210)	0.008^2^ (0.122)	0^1^ (na)	0.034^2^ (0.08)	0.09^1^ (0.300)	0.008^1^ (0.189)	0^1^ (na)		
Prairie	0.001^2^ (0.060)	0.03^2^ (0.047)	0.025^2^ (0.170)	0.021^1^ (0.132)	0^1^ (na)	0.052^1^ (1.240)	0^1^ (na)	0^1^ (na)	0^1^ (na)	0.001^2^ (0.075)		

Superscripts indicate statistical distribution used in generalized linear mixed model (link function), except for germination (A), which was consistently modelled as a binomial distribution (link = logit).

na = not applicable

^1^Poisson (link = log);

^2^Negative binomial (link = log);

^3^Gaussian (link = identity).


$$\begin{array}{l} h^{2}=\;\left(\;\frac{\left(2.5\;\times \;Var_{fam}\right)}{Var_{fam}+\;Var_{fam\;x\;block}+\;Var_{error}}\right), \end{array}$$

where *Var*_*fam*_ is the maternal family variance, *Var*_*fam*_ × *Var*_*block*_ is the variance attributed to the interaction between family and block, and *Var*_*error*_ is the error/residual variance. Heritability calculations from half-sibling analyses typically multiply the numerator, *Var*_*fam*_, by 4 to account for half-sibling relatedness (Lynch and Walsh, 1998; Campbell *et al.*, 2022). However, Ahrens *et al*. (2020) indicated that species with a putative mixed mating system, such as *G. triflorum,* should use a factor 2.5, which corresponds to a coefficient of relatedness of *r* = 1/2.5, or approximately a 30 % selfing rate. This predicted rate of selfing may be high but given that half-sibling analyses represents an upper limit to heritability, our calculation produces a conservative final estimate. While there has been no empirical work to date examining the breeding system of *G. triflorum*, related studies within the genus point towards a mixed mating system largely mediated by bee pollination (Vandepitte *et al.*, 2010; Ruhsam *et al.*, 2011, 2013; Volk *et al.*, 2022). Indeed, monoecious flowers characteristic of the species exhibit a high probability of self-fertilization (Ahrens *et al.*, 2020; Volk *et al.*, 2022). Furthermore, if not accounted for, the presence of inbred individuals may inflate heritability estimates. Therefore, we follow the estimates of heritabilities of Ahrens *et al*. (2020). Finally, we estimated coefficients of additive genetic variance (CV_A_) following Houle (1992) as:


$$\begin{array}{l} CV_{A}=\;\sqrt{\frac{\left(2.5\;\times \;Var_{fam}\right)}{\bar{x}}}, \end{array}$$

where $\bar{x}$ is the is the mean trait value.

**Fig. 2.**
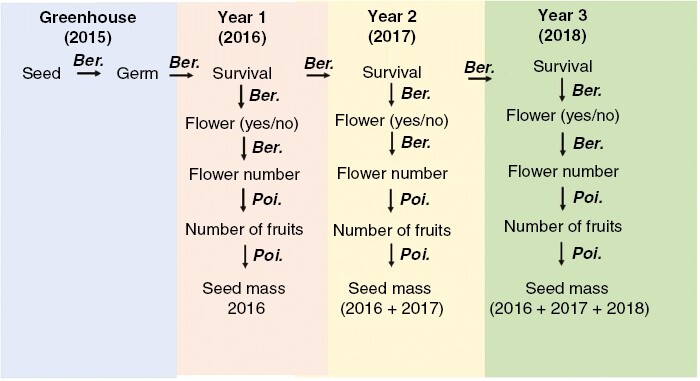
Graphical model used to estimate lifetime fitness for each plant in the common garden. Each node represents a fitness component and therefore response variable, and arrows represent conditional distributions. Probability of germination (Germ), flowering, and survival [0 or 1; Bernoulli (Ber.) distribution], and total number of flowers, total number of fruits and seed mass (Poisson). Seed mass for each year was calculated as the sum of seed mass for the current year and all previous years of the experiment.

Over the course of our experiment, total fitness was estimated using aster models (Geyer *et al.*, 2007; Shaw *et al.*, 2008). Mean fitness was estimated for each year of the study for each of the Great Lakes alvars, Manitoba alvars, and prairie regions. The graphical model for fitness (Fig. 2) included individual plant assessments of emergence, survival across years, and survival to flowering modelled as Bernoulli distributions. For flowers, fruits and seed mass production was modelled as a Poisson distribution. The terminal fitness node of seed mass was included as the cumulative seed mass for the current and previous year, for each given year of study. We followed the recommendation of Bolker *et al.* (2009) and Geyer *et al.* (2013) and treated block as a fixed effect. Thus, we avoided the computational issues associated with the relaxation of Gaussian assumptions for random effects that relate to all generalized linear models, including aster models, and were explicitly motivated by the need to model cases that did not meet these specific assumptions. For each region and year combination, mean fitness was represented by the median block estimate for fitness (and standard error).

Fitness landscapes, characterized with aster, were calculated to describe selection on individuals in the common garden, while accounting for historical selection in source populations through the addition of population-specific environmental summaries. To analyse the expression of early phenological transitions (time to emergence in 2016) and life-history trait variation (number of flowers produced in each year) in the common garden environment, year-specific aster models were used to calculate cumulative fitness landscapes. Time to emergence and annual variation in flower production were assessed as they exhibited a relatively high proportion of heritable variation (Table 2), with increased potential to respond to selection. Selection associated with the environment of origin for each population was included in the aster fitness landscape models using the first principal component that differentiated population climate as established in Yoko *et al.* (2020), based on 26 average annual climate variables estimated from ClimateNA (Wang *et al.*, 2016). We estimated selection across populations planted within the common garden using the first axis of climatic variation, PC1 (43 % of total variation), which previous research suggests largely follows a gradient in seasonal water availability, and a second axis of climatic variation, PC2 (~27 % of total variation), which largely follows a temperature gradient (Yoko *et al.*, 2020). To allow for correlational selection in fitness landscapes, aster models included the cross-products between the traits (days to emergence or number of flowers) and the first principal component following Geyer and Shaw (2010). For each fitness landscape, a summary of climatic variation (PC1) of origin was used as a predictor variable for yearly trait observations within the common garden, including days to emergence (2016) and annual number of flowers produced (2016–18). Selection was represented by distinct fitness contours associated with the year of trait observation. The steepness of the fitness landscape topography, i.e. the magnitude of selection, is reflected by the proximity and the increment of change between contour lines. We superimposed observed individual-plant phenotypes on fitness landscapes to show the distribution of individuals sourced from each region within the estimated selection surfaces.

## RESULTS

### General patterns of fitness expression and phenology

Plants sourced from the two alvar habitats exhibited similar and greater initial success in emergence relative to plants sourced from prairie habitats (*χ* = 278.43, *P* < 0.0001). The proportions of seedlings that emerged from planted Great Lakes alvar (972 of 1312 = 0.741) and Manitoba alvar populations (180 of 239 = 0.753) were greater than those of seedlings from prairie populations (298 of 790 = 0.377). Similar differentiation between alvar and prairie regions were observed for early phenological expression: the mean ± standard error for number of days between planting to emergence (Great Lakes alvars, 10.8 ± 0.08; Manitoba alvars, 11.6 ± 0.30; prairies, 14.4 ± 0.33; all *t* > 3.38, *P* < 0.001), planting to the production of true leaves (Great Lakes alvars, 18.4 ± 0.09; Manitoba alvars, 18.9 ± 0.31; prairies, 21.5 ± 0.34; all *t* > 1.99, *P* < 0.05), and days to first flower (Great Lakes alvars, 271.1 ± 6.61; Manitoba alvars, 271.1 ± 6.6; prairies, 266.1 ± 5.44; all *t* < 0.85, *P* > 0.390). Interestingly, early similarities between populations sourced from different alvar regions disappeared with later expressions of phenology. For example, the mean ± standard error number of flowers produced in the second year of the study (when more plants flowered to permit comparison) diverged based on habitat origin (Great Lakes alvars, 11.5 ± 0.28; Manitoba alvars, 5.2 ± 0.37; prairies, 3.1 ± 0.32; all *t* < −8.87, *P* < 0.0001). Finally, total mean ± standard error seed mass (accounting for differing numbers of plants per region) differed widely across all three regions (Great Lakes alvars, 1212.9 ± 42.05 mg; Manitoba alvars, 311.8 ± 35.53 mg; prairies, 367.3 ± 32.74 mg; all *t* < −6.43, *P* < 0.0001).

### Heritability and evolvability: fitness expressions and phenology

Heritability for fitness expressions (mean *h*^2^ = 0.109, Table 2A) were generally greater than those estimated for phenological traits (mean *h*^2^ = 0.052, Table 3A). For fitness expressions, heritability ranged from 0 to 0.317, with heritability of fitness expressions from earlier life-history stages greater relative to later life-history fitness expressions. Similarly, the heritability for phenological traits ranged from 0 to 0.202, with a greater contribution of heritable genetic variation to early phenological transitions relative to later phenological transitions. Keeping with these estimates, evolvabilities (coefficient of additive genetic variation) were also generally larger for early life-history fitness expressions (Table 2B) but were generally non-existent for phenological traits (Table 3B). Moreover, and in accordance with patterns of early phenological traits, the estimates of narrow-sense heritability for the number of days to emergence were relatively consistent between the two alvar regions (Great Lakes, 0.257; Manitoba, 0.154) when compared with populations from the prairie region (0.026). Only two individuals from the Manitoba alvar and prairie habitat types produced fruit in 2016. Therefore, we did not attempt to estimate heritability or evolvabilities for fruit set or seed mass in 2016 from these two regions.

**Table 3.** (A) Narrow-sense heritability and standard error and (B) evolvability (coefficient of additive genetic variation) of *G. triflorum* phenology events grown in a common garden.

Region	Number of days to emergence	Planting to bolting 2017	Planting to bolt 2018	Planting to first flower 2016	Planting to first flower 2017	Planting to first flower 2018	Planting to fruit 2017	Planting to fruit 2018
(A) Narrow-sense heritability								
Great Lakes alvar	0.257 (0.021)	0.059 (0.045)	0.011 (0.012)	0.002 (0.001)	0.202 (0.003)	0.197 (0.057)	0.000 (0.000)	0.059 (0.018)
Manitoba alvar	0.154 (0.070)	0.001 (0.015)	0.013 (0.037)	0.000 (0.000)	0.136 (0.054)	0.086 (0.130)	0.010 (0.042)	0.013 (0.026)
Prairie	0.026 (0.016)	0.001 (0.012)	0.001 (0.008)	0.000 (0.000)	0.000 (0.000)	0.000 (0.000)	0.000 (0.000)	0.013 (0.021)
(B) Evolvability								
Great Lakes alvar	0.111	0.004	0.001	0.000	0.000	0.003	0.000	0.001
Manitoba alvar	0.147	0.001	0.001	0.000	0.002	0.003	0.001	0.001
Prairie	0.049	0.000	0.001	0.000	0.000	0.000	0.000	0.001

More generally, heritability estimates for fitness expressions and phenological traits exhibited consistent variation among habitat types. Populations from both alvar regions (Great Lakes and Manitoba alvars) expressed a larger proportion of heritability relative to populations from the prairie region (Table 2).

### Mean fitness and selection

**Fig. 3.**
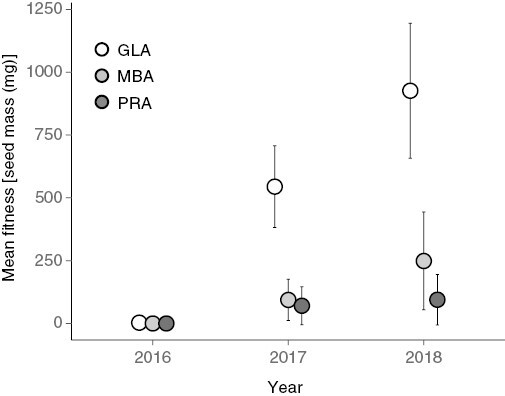
Estimated mean fitness and standard errors for plants from Great Lakes alvars (GLA), Manitoba alvars (MBA) and prairie (PRA) source populations across three successive years.

Mean fitness, determined as annual cumulative seed mass, varied widely across regions and years. The region of seed origin and year of study (and their interaction) had significant effects on the fitness of *G. triflorum* plants in the common garden (all test deviance > 82.09, all *P* < 0.0001). Fitness represented by the median block estimate (and standard error) was consistently higher in plants originating from the Great Lakes alvar region for all three years of study. This contrasted with plants from the Manitoba alvar and prairie regions, which produced almost no seed until the second year of study (Fig. 3), precluding fitness comparisons among regions in 2016. Plants from prairie populations consistently exhibited reduced fitness relative to Great Lakes alvar populations, with Manitoba alvar plants intermediate to these two regions. Region-specific estimates of fitness increased throughout the course of our study. Whereas the rate of yearly fitness increase was much greater in Great Lakes compared with Manitoba alvars in the first two years of our study, the rate of annual fitness increase began to converge across regions in the third year.

**Fig. 4.**
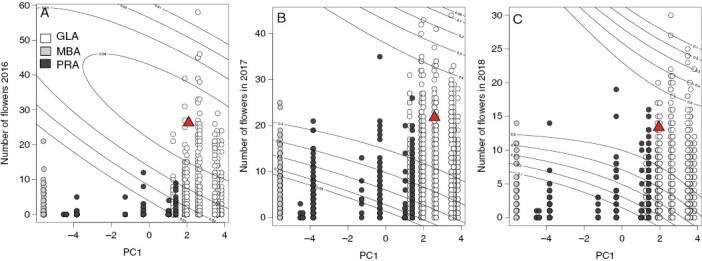
Fitness landscapes for the total number of flowers produced and a principal component describing a moisture gradient based on climate of origin for seed source populations from Yoko *et al.* (2020) over three consecutive years (A, 2016; B, 2017; C, 2018) in the common garden. Points denote observed numbers of flowers and line contours indicate fitness contours determined with aster models following Geyer and Shaw (2008). Great Lakes alvars (GLA), Manitoba alvars (MBA) and prairie (PRA) source populations and identified fitness optimum (red triangle) are indicated.

Calculation of fitness landscapes identified fitness optima for days to emergence in 2016 (Supplementary Data Fig. S1) and number of flowers for all three years of the study (Fig. 4A–C) with a principal component of source-population environmental variation. This principal component primarily described a soil moisture gradient associated with population origin (Yoko *et al.*, 2020). Regardless of trait–PC1 combination, selection was weak in 2016 with small fitness changes across fitness intervals. In subsequent years, selection on flower number and PC1 became stronger, and the range of optimal flower number–PC1 combination became successively narrower across years (Fig. 4). Consistent with estimates of mean fitness, individuals from the Great Lakes regions were consistently distributed closest to the fitness optima in each year of study.

## DISCUSSION

Populations of *G. triflorum* from alvar and prairie habitats have experienced varying predictability of seasonal cues that have resulted in differing programmes of habitat-specific selection. Varying the predictability of selection was expected to result in different degrees of genetic and environmental (i.e. plastic) influence on life-history traits. Our prediction of a stronger genetic basis in life-history traits in plants from predictable alvar habitats was supported, with early life-history traits relatively more heritable in plants from both habitat types. Unexpectedly, fitness estimates of plants from alvar habitats were consistently higher than that of prairie plants, placing alvar populations closer to the fitness optimum in the prairie common garden environment. This unexpected result may be explained by enhanced germination success and water-use efficiency in plants from alvar habitats. Below we elaborate on these findings and discuss the consequences of environmental predictability in local adaptation.

### Heritability and evolvability in the common garden

The potential for traits to respond to selection is in part dependent on the degree of standing genetic variation for traits under selection. Overall, individuals of *G. triflorum* from predictable alvar habitats exhibited larger estimates of heritability in both fitness expressions (Table 2) and phenology (Tables 2 and 3) compared with plants from unpredictable prairie habitats. In particular, the probability of emergence and total number of flowers produced in the common garden, features important to fitness, were higher for alvar compared with prairie plants. The timing of emergence has important consequences for later life-cycle events (e.g. flowering onset), and supports the prediction of earlier traits being under relatively stronger genetic influence (Price and Schluter, 1991), especially in environmentally unpredictable prairie habitats (Leung *et al.*, 2020). Further, heritability of the phenological aspect of emergence (number of days to emergence, Table 3) was 1.5–2 orders of magnitude greater in plants from alvars compared with prairie habitats. Importantly, this has implications for estimates of evolvability, as the CV_A_ values for timing of emergence for plants from alvar habitats were twice that of plants from prairie habitats. Therefore, the probability and timing of emergence may be twice as responsive to selection in plants from alvars compared with prairies. Optimal timing of emergence would be crucial in alvar habitats to ensure sufficient time for reproduction (i.e. flowering onset) before the predictable summer drought (e.g. Franks *et al.*, 2007). Interestingly, the probability of emergence, which exhibits the highest degree of evolvability, was comparable across regions (Table 3), suggesting that although the heritability of emergence in prairie environments is reduced, the potential response to selection is similar across habitats of origin.

### Genetic and environmental contributions to phenology

The timing of seedling emergence is an ecologically important aspect of phenology and closely associated with fitness. When a seedling emerges determines the future environment experienced during growth, reproduction and seed dispersal (Evans and Cabin, 1995), and has thus been interpreted as a form of niche construction (Donohue, 2005). Therefore, local adaptation should include a strong heritable basis for the timing of emergence. Our estimates of heritability and evolvability were greater in plants from alvar compared with prairie environments. With predictable seasonal extremes of flooding and drought in alvars (Reschke *et al.*, 1999), individuals experience restricted time for reproductive cycles. Therefore, a strong heritable basis for the timing of emergence should be prevalent in alvar environments to maximize the amount of time for reproductive effort before the onset of late-season dormancy. In contrast, prairie environments lack this consistent signal of environmental coordination, resulting in a more variable emergence schedule (Yoko *et al.*, 2020; Volk *et al.*, 2022). Greater plasticity in timing of emergence is reflected in overall lower estimates of heritability in plants from prairie populations (Table 3).

Alternatively, the difference in the magnitude of heritability between Great Lakes alvar and prairie plants could be attributed to variation in the demography of Great Lakes alvar populations. These populations are generally large and exhibit increased density within similar spatial extents relative to prairie populations (Hamilton and Eckert, 2007). Further, alvar habitats typically experience reduced interspecific competition due to the unique environmental features that support the persistence of select flora on these habitats (Partel *et al.*, 1998). This contrasts with prairie habitats, where populations may also be fragmented, but forbs often experience increased competition with native and invasive grasses (Dickson and Busby, 2009).

### Consequences of predictable and unpredictable environments

Provided that sufficient standing genetic variation exists, predictably varying environments present populations with a consistent pattern of selection resulting in an evolutionary response. However, consistent and predictable selection need not necessarily erode additive genetic variation for traits as traditionally conjectured for traits presumed to be closely associated with fitness (Roff and Mousseau, 1987; McFarlane *et al.*, 2014). Provided that sufficient annual variation in the predictable environmental cue exists, optimal responses to this cue will vary across years. Therefore, fluctuation in the direction and magnitude of selection may maintain appreciable additive genetic variation (Bell, 2010). We observed such a pattern in the timing of emergence and flowering regardless of habitat of origin (Table 3).

Given the importance of the timing of emergence to subsequent life-history events, a strong genetic basis could provide a consistent starting point for life-history events. However, greater environmental variation associated with later life-history events could cumulatively impact the evolutionary trajectory of phenotypic traits. Indeed, later life-history expressions have been predicted to exhibit relatively reduced estimates of heritability (Price and Schluter, 1991). Therefore, the degree of plasticity for post-emergence life-history events would be much greater than during early life-cycle events. In our study, estimates of heritability and evolvability for the timing of emergence were consistently larger than later life-history events regardless of habitat of origin. Heritability for the timing of flowering was reduced, but still appreciable compared with the remaining life-history events. This pattern matches our predictions that heritable variation is of greater importance in early life-history events. During later events, environmental variance is greatest and therefore plasticity will have a relatively larger effect on phenotypic variance. This pattern implies some continuum of the relative effects of heritable and environmental variance on phenotypic variation. Such a continuum would result in an intermediate life-history expression that has an approximately equal proportion of heritable and plastic determinism. Given the intermediate estimates of heritability for the timing of flowering (across years; Table 3), this life-history event may represent such an intermediate. Indeed, this phenological signpost has been shown to exhibit similar magnitudes of narrow-sense heritability in multiple species (Wheelwright, 1985; Mitchell and Shaw, 1993; Geber and Griffen, 2003; Burgess *et al.*, 2007; Goncalves-Vidigal *et al.*, 2008), and other studies confirming adaptive plasticity in the timing of flowering (Donohue *et al.*, 2000; Jimenez-Ambriz *et al.*, 2007). Plasticity in flowering time seems to be more common than not (Levin, 2009). For example, Ensing and Eckert (2019) report a gradient of plasticity that resulted in the timing of flowering in *Rhinanthus minor* transplanted along a range of altitudes that plastically shifted flowering time to match those of new conspecifics. This midpoint life-history event may represent the transition when environmental variation begins to exceed heritable variation in determining phenotypic variation, and ultimately population fitness.

### Interannual patterns of heritability

Estimates of heritability can vary across years for the same trait in the same locality. However, if heritability estimates are constant across environments some ability to predict phenotypes as a response to selection exists. For example, Young *et al.* (1994) determined consistent heritability of floral traits in *Raphanus sativus* in three different environments, indicating the absence of a genotype-by-environment interaction. Therefore, the expectation is that selection acting on these traits would result in environment-specific changes in phenotypes. In contrast, our results suggest that, in general, heritability for expressions of fitness (flower number, fruit number and seed mass) of plants from alvar habitats decreased over time, supporting the prediction from Price and Schluter (1991) that the cumulative exposure to environmental variability limits the estimate of narrow-sense heritability. The same traits in plants from prairie environments exhibited very little variation across years, and overall negligible estimates of heritability. Similarly, heritability estimates for fitness expressions were overall smaller than those for phenology traits, regardless of habitat origin.

The expression of additive genetic variation is dependent on local environmental conditions (Hoffmann and Merila, 1999; Sheth *et al.*, 2018) and may change under unfavourable or stressful conditions (Schlichting, 2008; Emery and Ackerly, 2014).Therefore, the potential rate of the response to selection will depend on the environment-specific expression of additive genetic variation. For example, Torres-Martinez *et al.* (2019) found greater short-term potential for adaptation during stressful drought (La Niña) years compared with more favourable wet (El Niño) years in an experimental precipitation gradient in *Lasthenia fremontii.* The temporal availability of water in alvar habitats likely imposes severe drought stress that accompanies periods of drought (Rosén, 1995; Schaefer and Larson, 1997; Lundholm and Larson, 2003). Individuals in our study from alvar communities likely experienced drought stress during later life-history stages, predicting higher expressions of additive genetic variance and greater heritability (but see Blows and Sokolowski, 1995; Charmantier and Garant, 2005). This may seem in conflict with the above discussion of later life-history events exhibiting smaller estimates of heritability, as found in our study. However, enhanced additive genetic variance does not necessarily equate to larger estimates of heritability, but rather depends on the proportion of environmental variation associated with traits. Therefore, the cumulatively larger environmental variance associated with later life-history events occurring throughout times of drought could reduce heritabilities regardless of enhanced expression of additive genetic variance. Our estimates of low heritability and evolvability of life-history events during these periods of stress suggest that environmental variance could hamper an adaptive response to selection (Falconer and Mackay, 1996; Levin, 1998; but see Ghalambor *et al*., 2007).

### Mean lifetime fitness and fitness landscapes

#### Mean lifetime fitness.

Mean fitness, as determined through aster models, relates directly to per capita rates of population increase, and therefore population sustainability and growth. Interestingly, plants from Great Lakes alvar populations consistently exhibited the highest fitness estimates in the common garden environment across all three years (Fig. 3). This was unexpected as alvar habitats differ markedly, especially in terms of water availability, from prairie habitats. Even plants sourced from prairie environments near the common garden did not perform nearly as well as plants from either Manitoba alvar or Great Lakes alvar populations. The large discrepancy in fitness may be attributed to low germination success in prairie plants (37.7 %) compared with plants from Great Lakes (74.1 %) and Manitoba (75.3 %) alvars. Overall, fitness was low in the first year of our study, as plants became established in the common garden and with relatively fewer plants flowering compared with subsequent years. In the remaining two years of the study, as fitness increased across all regions of origin, the difference in fitness among regions increased while maintaining the same pattern of fitness expression (Fig. 3). Therefore, the pattern of plants from Great Lakes alvar populations exhibiting higher fitness in the common garden was not a short-term artefact of establishment.

The expression of fitness, much like the expression of additive genetic effects, is dependent on the environment. When genotypes are moved from their home range to a novel environment, fitness may decrease as a sign of local adaptation (Hereford, 2009; Garrido *et al.*, 2012), increase (e.g. Sheth *et al.* 2018) or remain constant (Galloway and Fenster, 2000). In our study, the movement of genotypes that evolved under Great Lakes alvar environments to the prairie environment of the common garden resulted in an increase in fitness. Yoko *et al.* (2020) detected trait enhancements associated with water-use efficiency, among others, in plants from alvars compared with prairie environments. Plants originating from alvars would experience greater water availability in the prairie environment of the common garden, where thick rich soils mitigate unpredictable fluctuations in water availability (Risser *et al.*, 1981; Anderson, 2006). Therefore, enhanced water-use efficiency of alvar plants could provide a physiological advantage over prairie plants that would not historically experience predictable seasonal drought conditions. Finally, ecological differences rather than physical distances are likely more important in determining fitness in our common garden. However, to fully evaluate the preadaptation of alvar plants would require a reciprocal transplant experiment with both alvar and prairie populations.

#### Fitness *landscapes.*

 Annual changes in mean fitness across source habitats corresponded to changes in the magnitude of selection as determined through fitness landscapes. Total flower number is commonly found to be under selection (Harder and Johnson, 2009) and therefore closely linked to fitness. We found moderate selection on the total number of flowers produced along an axis of environmental variation (moisture gradient) for source populations. The magnitude of selection along the axis of environmental variation represents the difference in environment between source populations and the common garden, describing the discrepancy between fitness optima across source populations and the novel common garden environment. Thus, the change in selection along the environmental gradients represents the degree of maladaptation following introduction to a novel environment.

The location of fitness optima consistently tracked mean fitness for each source population habitat type (Figs 3 and 4). Fitness across all source populations was modest in the first year of study with few individuals reproducing. Consequently, the fitness landscape (Fig. 4A) was relatively flat with a wide but shallow plateau surrounding the optima. In the two remaining years of the study, the topography of the landscapes became steeper, indicating stronger selection, with successively more plants occupying the region around the fitness optima. Regardless of year, plants from the Great Lakes alvars were consistently closer to the fitness optima. Alvar habitats experience consistent extremes in water availability with annual late-season desiccation (Catling and Brownell, 1995). Lack of water availability favours selection for plants with reduced water potential and enhanced water-use efficiency (Craine and Dybzinski, 2013). Thus, plants originating from predictably water-stressed environments like alvars could express release from water-use constraints, effectively enhancing fitness in response to increased water availability within the prairie common garden environment. In contrast, plants from prairie populations did not experience such a drastic change in water availability. Therefore, and unexpectedly, the degree of environmental maladaptation in the common garden environment was greater for plants from prairie populations than for plants from alvar populations.

Over successive years, the breadth of the fitness optimum increased along the axis of environmental variation but was relatively constrained along the axis of total flower production. Given the relatively high heritability of flower number across all three years of the study (Table 2), the limited plasticity in flower number may be responsible for this constraint. In contrast, enhanced plasticity for water-use traits has been associated with improved survival and seed production (Nicotra and Davidson, 2010). Therefore, the more predictable seasonal changes in water availability in alvar habitats may promote enhanced plasticity in water use traits, whereas flower number would remain relatively constant within a habitat type. However, across habitat types, total flower production was greater in plants from alvars with predictable changes in water availability compared with prairie habitats. Regardless of habitat origin, the degree of plasticity associated with flower number was restricted compared with that of water availability. Therefore, the restricted plastic response of flower production would impose a strong limitation on fitness and local adaption in the common garden environment.

Total fitness is the cumulative expression of an individual’s fitness components across its life cycle, from emergence and survival to the total number of offspring produced. Individual fitness expressions are the results of genetic and environmental (plastic) effects, as well as their interaction. We observed that the independent and interactive effects of these components vary continuously across life histories. The relative degree of genetic and environmental contributions to trait variation provides a new perspective to the dynamics of life-history traits. We found a shift from genetic to greater environmental effects across successive years, allowing plants farthest from the fitness optimum to traverse the fitness landscape and increase proximity to the fitness optimum. Plants sourced from predictable alvar environments started closest to and maintained proximity to the fitness optimum across years. Whereas plants from unpredictable prairie environments were initially farthest from the fitness optimum, these plants were able to quickly approach the fitness optimum. Overall, these results highlight the importance of environmental variation in facilitating movement across the fitness landscape, and genetic variation in maximizing fitness near the optimum.

### Conclusions

Maximizing fitness across coordinated life-history events requires synchronous phenotypic responses to environmental cues. The challenge to maximize fitness is made more complex when seasonal cues are unpredictable and projected to become less predictable under global change. We show that predictable seasonal cues in alvar environments have led to substantial genetic control over early life-history traits, such as probability of and days to emergence, but plasticity for later life-history traits that enable longer-term fitness tracking. In contrast, individuals from prairie habitats, which are characterized by less predictable seasonal cues, exhibited overall reduced heritability for the same traits. This reduced heritability may limit the ability of a perennial species to traverse fitness landscapes across generations. Therefore, a continuum of relative heritable-to-environmental (plastic) effects may be optimized by natural selection to match specific habitat types (e.g. predictable or unpredictable habitats). Finally, variance in the genetic or environmental contribution to phenological and life-history trait variation may have substantial influence on fitness, particularly when considering the capacity of individuals to maintain fitness across environments as seasonal cues shift under global change.

## SUPPLEMENTARY DATA

Supplementary data are available online at https://academic.oup.com/aob and consist of the following. Figure S1: fitness landscapes for the number of days to emergence from planting and a principal component describing a moisture gradient in source populations from Yoko *et al*. (2020) for the first year of study (2016). Points denote observed numbers of days to emergence and line contours indicate fitness contours determined with aster models following Geyer and Shaw (2008). Great Lakes alvars (open circles), Manitoba alvars (light grey circles), and Prairie (dark grey circles) source populations and identified fitness optimum (red triangle) are indicated.

mcad100_suppl_Supplementary_Figure_S1

## ACKNOWLEDGEMENTS

The authors thank Jon Sweetman, Chad Stratilo, Mary Vetter, Rebekah Neufeld, Tyler Stadel, Steve Travers, and Nature Conservancy of Canada for help with initial field sampling, and Stephen Johnson, Nick Hugo, Alexis Pearson, Zoe Portlas, Naomi Hegwood, Kate Volk, Jeff Kittilson, and Storm Nies for assistance in the field. Particular thanks to Tony Bormann (MSUM Science Center) for logistical support.

## FUNDING

This work was supported by a new faculty award from the office of North Dakota Experimental Program to 508 Stimulate Competitive Research (ND-EPSCoR NSF-IIS-1355466) to J.A.H.
