# Supplementary figures and images for: Chasing the fitness optimum: temporal variation in the genetic and environmental expression of life-history traits for a perennial plant
Source: Ann Bot. 2023 Jul 26;132(7):1191–204. doi: 10.1093/aob/mcad100 (PMC10902883; doi:10.1093/aob/mcad100)

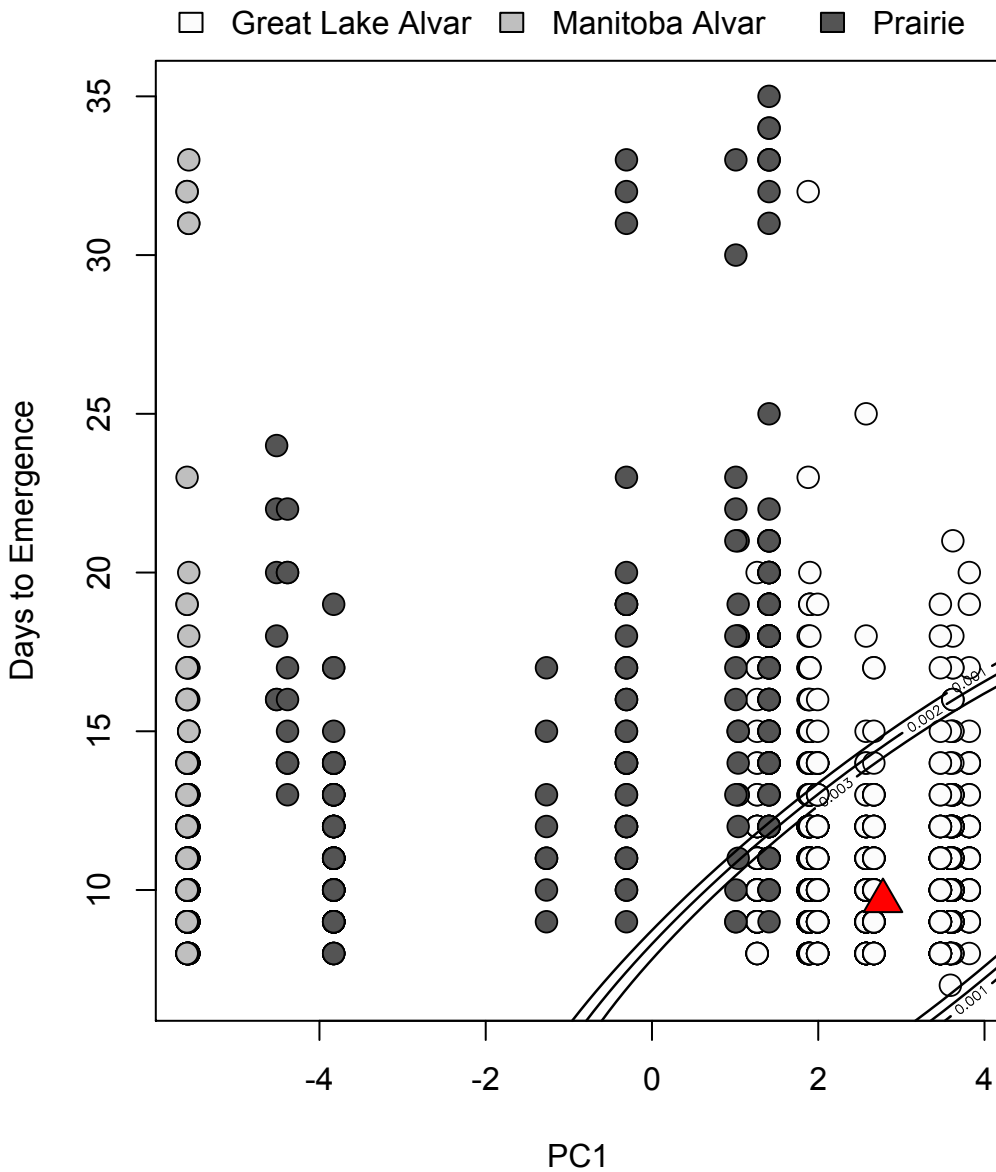

Supplement: mcad100_suppl_Supplementary_Figure_S1 [file mcad100_suppl_supplementary_figure_s1.pdf]
